# Outcomes of Moderate-to-Severe Traumatic Brain Injury in a Low-Resource Setting

**DOI:** 10.1001/jamanetworkopen.2026.28403

**Published:** 2026-08-11

**Authors:** Kantesh Kumar, Simran Motwani, Komal Abdul Rahim, Muhammad Muneeb Khan Bozai, Sheza Hassan, Tanweer Ahmed, Saima Mushtaq, Huba Atiq, Yasir Shafiq, Shahzad Shamim, Adil Haider, Junaid Razzak

**Affiliations:** 1 Centre of Excellence for Trauma and Emergencies, The Aga Khan University, Karachi, Pakistan; 2 Department of Emergency Medicine, Weill Cornell Medicine, New York, New York; 3 Department of Neurosurgery, Jinnah Postgraduate Medical Centre, Karachi, Pakistan; 4 Department of Emergency Medicine, Jinnah Postgraduate Medical Centre, Karachi, Pakistan; 5 Department of Surgery, The Aga Khan University, Karachi, Pakistan

## Abstract

**Importance:**

No study from a low- and middle-income country (LMIC) has reported long-term mortality and quality of life (QOL) after moderate to severe traumatic brain injury (msTBI), highlighting its burden and recovery trajectories.

**Objectives:**

To measure short- and long-term mortality and QOL among patients in an LMIC with msTBI and to identify factors associated with outcomes.

**Design, Setting, and Participants:**

This cohort study analyzed prospectively collected data of patients with msTBI from December 1, 2021, to May 31, 2024, from a multicenter trauma registry of all injured patients from 2 tertiary care centers in Karachi, Pakistan. Adult patients (aged ≥18 years) with a diagnosis of msTBI (Glasgow Coma Scale [GCS] score of ≤12) enrolled in the registry and had recorded outcomes at 1, 3, 6, and 12 months.

**Exposure:**

Moderate TBI (GCS score of 9-12) or severe TBI (GCS score ≤8 or intubated patients with recorded GCS).

**Main Outcomes and Measures:**

The primary outcome was mortality (inpatient, follow-up, and cumulative), and the secondary outcome was QOL.

**Results:**

Of the 819 patients (median age, 38 years [IQR, 26-54 years]; 689 men [84.1%]), 368 (44.9%) had moderate TBI and 451 (55.1%) had severe TBI; 158 patients (19.3%) were lost to follow-up at 12 months. Road traffic crashes were the most common cause of TBI (607 [74.1%]). Inpatient mortality was 22.9% among patients with moderate TBI (80 of 349) and 68.9% among those with severe TBI (295 of 428). For patients discharged alive, 8.2% with moderate TBI (30 of 368) and 5.8% with severe TBI (26 of 451) died within 1 month. Cumulative mortality at 12 months was 59.4% among patients with moderate TBI and 87.8% among those with severe TBI. Older age (adjusted hazard ratio [AHR], 1.01 [95% CI, 1.01-1.02] per additional year of age), TBI severity (AHR, 2.67 [95% CI, 2.24-3.18]), and having no surgical intervention (AHR, 1.43 [95% CI, 1.18-1.74]) were significantly associated with higher 12-month mortality. Patients with severe TBI had poor QOL reported across all domains compared with those with moderate TBI.

**Conclusions and Relevance:**

In this cohort study of patients in Pakistan with msTBI, mortality was high, particularly among those with severe TBI, and most posthospital deaths occurred within the first month after hospital discharge. Older age, no surgical intervention, and greater injury severity were associated with higher mortality, highlighting the need for improved acute and postdischarge care in resource-limited settings.

## Key Points

**Question:**

What are the patterns of short- and long-term mortality and quality of life (QOL) among patients with moderate to severe traumatic brain injury (TBI) in a low- and middle-income country?

**Findings:**

This cohort study assessed inpatient and 1-, 3-, 6-, and 12-month postdischarge mortality and QOL among 819 patients with moderate to severe TBI. Inpatient mortality was 22.9% for moderate TBI and 68.9% for severe TBI, with cumulative 12-month mortality of 59.4% and 87.8%, respectively; severe TBI was associated with poorer QOL across all domains.

**Meaning:**

This study suggests that moderate to severe TBI is associated with high mortality and long-term disability, underscoring the need to strengthen trauma care systems and improve acute and postdischarge management.

## Teaser

This cohort study measures short- and long-term mortality and quality of life among patients in Pakistan with moderate to severe traumatic brain injury and identifies factors associated with outcomes.

## Introduction

Traumatic brain injury (TBI) is a global health concern, with head injuries being the most significant risk factor for death and long-term disability among all injury types.^1^ Often referred to as a silent epidemic,^2^ an estimated 69 million individuals sustain a TBI annually, with low- and middle-income countries (LMICs) experiencing nearly 3 times more cases and twice the odds of mortality after severe TBI than higher-income countries (HICs).^1^ With an annual economic burden of $400 billion, TBI poses a major economic and societal challenge.^3,4^

TBI has been extensively studied in HICs, with substantial data on both short-term and long-term outcomes. Numerous studies from these regions highlight the recovery trajectory after moderate to severe TBI (msTBI). For instance, a prospective multicenter survey reported that favorable outcomes (defined as scores between 4 and 8 on the Glasgow Outcome Scale–Extended) were observed among 12.4% of patients with severe TBI and 41% of those with moderate TBI at 2 weeks after injury, which significantly improved to 52.4% of those with severe TBI and 75% of those with moderate TBI at 12 months of follow-up.^5^ Despite patients in HICs having better access to care and health care facilities in HICs being well equipped, these studies show that recovery after TBI is often incomplete and delayed, with quality of life (QOL) compromised in the long term.

Although TBI outcomes are well known in HICs, the outcomes in LMICs are either not known (eg, due to short-term follow-up) or show evidence of significant methodological flaws (such as loss to follow-up). A recent meta-analysis identified 41% higher mortality after severe TBIs in developing countries.^6^ Among the LMICs, data were reported only from Iran and India, with a total reported number of 430 patients. None of the published studies from LMICs have long-term posthospital outcomes.

A significant knowledge gap persists regarding long-term functional and cognitive outcomes among patients with msTBI in LMICs; addressing this gap is crucial for informing health system priorities in resource-limited settings. To our knowledge, our study is the largest multicenter study reporting long-term outcomes in one of the largest cohorts from an LMIC. Our study measures short-term and long-term mortality (inpatient and 1, 3, 6, and 12 months) and QOL among patients with msTBI and identifies factors associated with these outcomes.

## Methods

### Study Design and Setting

We analyzed data using a multicenter trauma registry. The registry collects data on patients admitted with injuries in 2 tertiary care centers in Karachi, Pakistan. Jinnah Postgraduate Medical Center is one of largest public institutions, with 2000 beds, and provides care to patients for free or at low cost. Aga Khan University Hospital is one of the largest private institutions, with 700 beds. Both are teaching hospitals that have 24/7 emergency departments with emergency medicine, general surgery, and neurosurgery residency training programs. Registry details have been previously published.^7^ The study was approved by the ethical review committee of Aga Khan University. Written consent was obtained from the patients for in-patient data collection by trained data collectors. Patients were also informed regarding the follow-up data collection at 1, 3, 6, and 12 months; verbal consent was obtained at each follow-up call. This study is reported in accordance with the Strengthening the Reporting of Observational Studies in Epidemiology (STROBE) reporting guideline for cohort studies.^8^

### Study Population

Adult patients (aged ≥18 years) with a diagnosis of msTBI (Glasgow Coma Scale [GCS] score of <13 or intubated with a recorded GCS) enrolled in the Trauma Registry and who had recorded outcomes at 1, 3, 6, and 12 months were included. eFigure 1 in Supplement 1 illustrates the data inclusion and exclusion pathway. Moderate TBI was defined as a GCS score of 9 to 12. Severe TBI was defined as a GCS score of 8 or less or intubation with recorded GCS score.

### Data Collection and Variables of Interest

Data were collected from December 1, 2021, to May 31, 2024, via a registry capturing sociodemographic variables, clinical variables, and long-term patient outcomes data through telephonic follow-up at 1, 3, 6, and 12 months by a trained data collector (T.A. and S. Mushtaq) using a structured guide in English and Urdu. Sociodemographic variables included age, sex, and educational status. Injury-related variables included the mechanism of injury (road traffic crashes, falls, assault, and others); Kampala Trauma Score (KTS; categorized as mild [9-10], moderate [7-8], and severe [<6]); and Modified Shock Index, calculated as heart rate divided by mean arterial pressure, to estimate mortality and need of resuscitation (classified as low risk [<0.70], normal risk [0.70-1.30], borderline high risk [1.31-1.69], and high risk [≥1.70]). The KTS is a composite trauma severity score that integrates physiological parameters (systolic blood pressure, respiratory rate, and neurologic status) with injury burden (Abbreviated Injury Scale score of ≥3) and patient age to provide an assessment of injury severity.^9^

Clinical variables included hospital ward (high dependence unit, intensive care unit, or ward), surgical intervention (yes or no), comorbidities (yes or no), neurosurgical procedures (craniotomy, craniectomy, or burr holes), and TBI diagnosis retrieved from the medical records (eg, contusions, subdural hematoma, and epidural hematoma). Patient outcomes included inpatient mortality, postdischarge follow-up mortality, and cumulative mortality (censored for loss to follow-up). Patient status at each follow-up interval was categorized into alive, dead, patients who died within that follow-up period, lost to follow-up, not eligible, and patients who were dead before this follow-up period. The secondary outcome was self-reported QOL, assessed using the Revised Trauma-Specific Quality of Life (RT-QOL) instrument. It measures QOL across 3 domains: (1) functional well-being, (2) emotional well-being, and (3) physical well-being and recovery.^10,11,12^

### Statistical Analysis

Descriptive analysis used frequencies and percentages for categorical variables; associations were tested using the χ^2^ test. Inpatient mortality was summarized using Kaplan-Meier curves stratified by TBI severity with log-rank testing. Cox proportional hazards regression identified independent factors associated with inpatient mortality; TBI severity was stratified due to proportional hazards assumption violation (Schoenfeld residuals). Patients with missing hospital discharge dates (n = 50) had their length of stay imputed using the cohort median length of stay. The final Cox proportional hazards regression model included age, gender, TBI severity (stratified), surgical intervention, comorbidities, and Modified Shock Index.

Postdischarge and cumulative mortality were analyzed using interval-censored semiparametric proportional hazard models. Deaths were entered as intervals [*L*, *R*], where *L* is last follow-up confirming survival, and *R* is first follow-up reporting death; patients lost to follow-up or alive at 12 months were right censored (*R* = ∞). Inpatient deaths in the cumulative mortality model were entered as degenerate intervals [*t*, *t*]. Survival curves were estimated using the Turnbull nonparametric maximum likelihood estimator, and group comparison used a regression-based likelihood ratio test. Cumulative mortality at each follow-up interval (1, 3, 6, and 12 months) was extracted from Turnbull nonparametric maximum likelihood estimator (NPMLE) curves overall and stratified by TBI severity. Standard errors for both interval-censored models were obtained via 1000 bootstrap samples (seed 2024). The proportional hazards assumption for the cumulative mortality model was assessed via complementary log-log plots of Turnbull NPMLE curves; TBI severity showed evidence of a time-varying effect, and the reported hazard ratio (HR) represents a time-averaged estimate. Unadjusted and adjusted HRs with 95% CIs are reported for all 3 models.

Loss to follow-up was characterized by comparing baseline and clinical characteristics between patients available for follow-up and those lost to follow-up (eTable 1 in Supplement 1). As loss to follow-up was nonrandom, the stabilized inverse probability weights (IPWs) derived from a logistic model of follow-up probability (factors: TBI severity, KTS, ward type, and intracranial diagnosis; area under the curve, 0.658) were applied to interval-censored models as a sensitivity analysis. Best-case mortality bounds were additionally estimated assuming all patients lost to follow-up survived at last contact, and worst-case mortality bounds were estimated assuming all patients lost to follow-up died at last contact (eFigure 2 in Supplement 1).

For the RT-QOL instrument, only patients alive at 12 months were included as the base population, and complete case analysis was performed at each follow-up interval. The responses agree, strongly agree, disagree, and strongly disagree were the only categories contributing to domain scoring; “Have no opinion” (value 3) and missing responses were excluded from both the numerator and denominator. Item direction was reversed where applicable. Domain scores were categorized as no symptoms (score = 0), mild (1-2), moderate (3-4), or severe (5-6). Patients with no valid responses for a domain at a given time point are reported as a separate count in the QOL table and excluded from all percentage calculations. All analyses were conducted using R software, version 4.6.0 (R Project for Statistical Computing). *P* < .05 was considered statistically significant.

## Results

A total of 819 patients (median age, 38 years [IQR, 26-54 years]; 689 men [84.1%] and 130 women [15.9%]) enrolled in the trauma registry met the inclusion criteria, with more than half aged 18 to 39 years (420 [51.3%]) and 295 (36.3%) with no formal education (Table 1). Of these, 368 patients (44.9%) had moderate TBI and 451 (55.1%) had severe TBI; 158 patients (19.3%; 97 of 368 patients [26.4%] with moderate TBI and 61 of 451 patients [13.5%] with severe TBI) were lost to follow-up (eTable 1 in Supplement 1).

**Table 1.** Distribution of Epidemiologic and Clinical Characteristics Among Patients With Moderate to Severe TBI

Characteristic	Patients, No./total No. (%)			*P* value
	Total (N = 819)	Moderate TBI (n = 368)	Severe TBI (n = 451)	
Age group, y				
18-39	420/819 (51.3)	184/368 (50.0)	236/451 (52.3)	.50
40-64	303/819 (37.0)	136/368 (37.0)	167/451 (37.0)	
≥65	96/819 (11.7)	48/368 (13.0)	48/451 (10.6)	
Sex				
Male	689/819 (84.1)	309/368 (84.0)	380/451 (84.3)	>.99
Female	130/819 (15.9)	59/368 (16.0)	71/451 (15.7)	
Educational status				
No formal education	295/813 (36.3)	135/366 (36.9)	160/447 (35.8)	>.99
Primary education	162/813 (19.9)	70/366 (19.1)	92/447 (20.6)	
Secondary education	197/813 (24.2)	90/366 (24.6)	107/447 (23.9)	
Intermediate	87/813 (10.7)	40/366 (10.9)	47/447 (10.5)	
Graduation and above	72/813 (8.9)	31/366 (8.5)	41/447 (9.2)	
Missing	6	2	4	
Cause of injury				
RTC	607/819 (74.1)	266/368 (72.3)	341/451 (75.6)	.60
Fall	164/819 (20.0)	81/368 (22.0)	83/451 (18.4)	
Assault	27/819 (3.3)	11/368 (3.0)	16/451 (3.6)	
Other	21/819 (2.6)	10/368 (2.7)	11/451 (2.4)	
RTC type				
Passenger (automobile or motorcycle)	111/593 (18.7)	54/257 (21.0)	57/336 (17.0)	.60
Motorcycle driver	370/593 (62.4)	159/257 (61.9)	211/336 (62.8)	
Automobile driver	11/593 (1.9)	4/257 (1.6)	7/336 (2.1)	
Pedestrian	101/593 (17.0)	40/257 (15.6)	61/336 (18.2)	
Unknown	226	111	115	
KTS				
Mild	219/811 (27.0)	207/367 (56.4)	12/444 (2.7)	<.001
Moderate	471/811 (58.1)	159/367 (43.3)	312/444 (70.3)	
Severe	121/811 (14.9)	1/367 (0.3)	120/444 (27.0)	
Missing	8	1	7	
Hospital ward				
High dependence unit	643/786 (81.8)	322/358 (89.9)	321/428 (75.0)	<.001
Intensive care unit	122/786 (15.5)	21/358 (5.9)	101/428 (23.6)	
Ward	21/786 (2.7)	15/358 (4.2)	6/428 (1.4)	
Missing	33	10	23	
Surgical intervention				
No	615/819 (75.1)	275/368 (74.7)	340/451 (75.4)	.80
Yes	204/819 (24.9)	93/368 (25.3)	111/451 (24.6)	
Procedure				
Craniotomy	83/204 (40.7)	40/93 (43.0)	43/111 (38.7)	.20
Craniectomy	55/204 (27.0)	19/93 (20.4)	36/111 (32.4)	
Burr hole	25/204 (12.3)	15/93 (16.1)	10/111 (16.1)	
TBI diagnosis				
Contusions	336/819 (41.0)	176/368 (47.8)	160/451 (35.5)	<.001
Subdural hematoma	297/819 (36.3)	116/368 (31.5)	181/451 (40.1)	
Epidural hematoma	165/819 (20.1)	89/368 (24.2)	76/451 (16.9)	
Skull fracture	94/819 (11.5)	39/368 (10.6)	55/451 (12.2)	
Intracerebral hemorrhage	89/819 (10.9)	23/368 (6.3)	66/451 (14.6)	
Diffuse axonal injury	43/819 (5.3)	12/368 (3.3)	31/451 (6.9)	
Pneumocranium	13/819 (1.6)	5/368 (1.4)	8/451 (1.8)	
Systolic blood pressure, mm Hg				
≥90	787/813 (96.9)	364/367 (99.2)	423/446 (94.8)	<.001
<90	26/813 (3.2)	3/367 (0.8)	23/446 (5.2)	
Missing	6	1	5	
Oxygen saturation, %				
≥90	730/814 (89.7)	356/365 (97.5)	374/449 (83.3)	
<90	84/814 (10.3)	9/365 (2.5)	75/449 (16.7)	
Missing	5	3	2	
MSI category				
Low (<0.70)	96/810 (11.8)	49/367 (13.4)	47/443 (10.6)	.04
Normal (0.70-1.30)	645/810 (79.6)	295/367 (80.4)	350/443 (79.0)	
Borderline high risk (1.31-1.69)	50/810 (6.2)	20/367 (5.5)	30/443 (6.8)	
High risk (≥1.70)	19/810 (2.4)	3/367 (0.8)	16/443 (3.6)	
Missing	9	1	8	

Abbreviations: KTS, Kampala Trauma Score; MSI, Modified Shock Index; RTC, road traffic crash; TBI, traumatic brain injury.

The cause of injury for most patients was road traffic crash (607 [74.1%]), most commonly as motorcycle riders (370 of 593 [62.4%]) (Table 1). A total of 75.1% of patients (615 of 819) received no surgical intervention, and 81.8% (643 of 786) were admitted to a high dependence unit). Most patients with moderate TBI had a mild (56.4% [207 of 367]) or moderate (43.3% [159 of 367]) KTS, while most patients with severe TBI had a moderate (70.3% [312 of 444]) or severe (27.0% [120 of 444]) KTS. More patients with moderate TBI than severe TBI were admitted to the high dependence unit (89.9% [322 of 358] vs 75% [321 of 428]), whereas more patients with severe TBI than moderate TBI were admitted to the intensive care unit (23.6% [101 of 428] vs 5.9% [21 of 358]). Contusions were the most common diagnosis among patients with moderate TBI (47.8% [176 of 368]), and subdural hematoma was the most common diagnosis for those with severe TBI (40.1% [181 of 451]).

**Figure 1.**
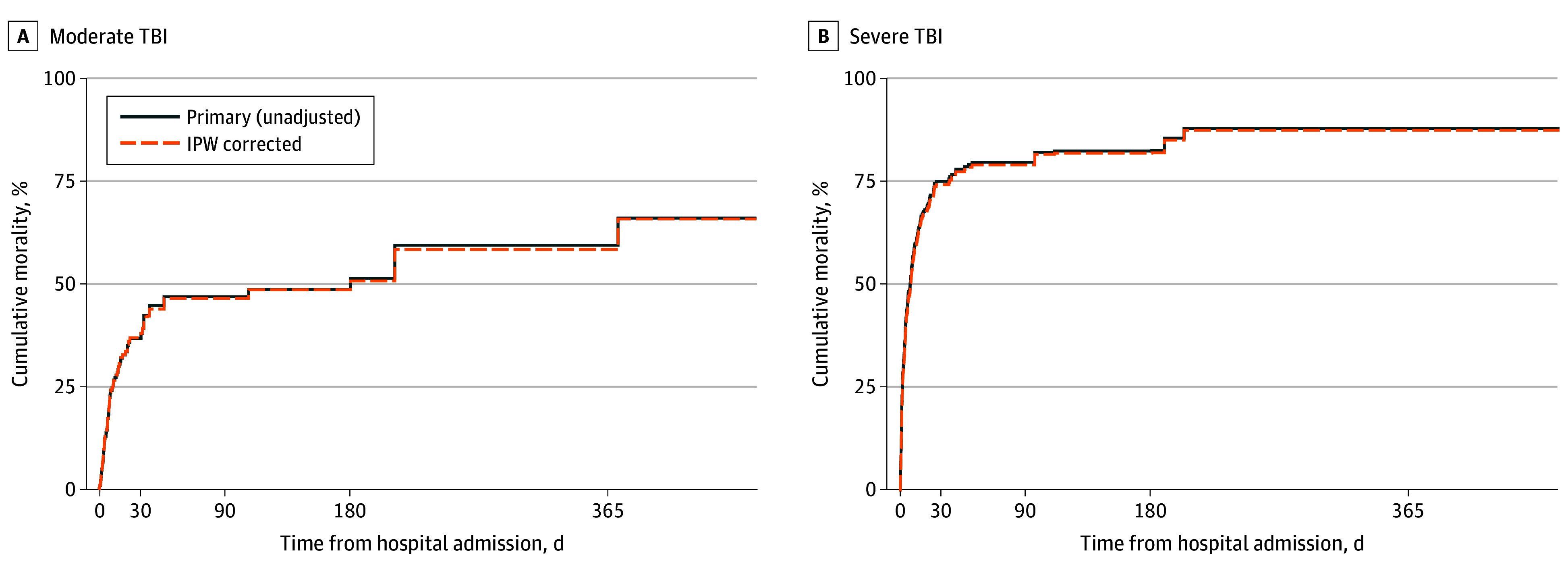
Line Graphs Showing Turnbull Nonparametric Cumulative Mortality Curves for Moderate and Severe Traumatic Brain Injury (TBI) Cumulative mortality from hospital admission estimated using Turnbull nonparametric maximum likelihood estimator, stratified by TBI severity. Primary (unadjusted) and inverse probability weighted (IPW)–corrected estimates are overlaid; curves differ by 1.1 percentage points or less, confirming robustness to nonrandom loss to follow-up. IPW-corrected estimates are the primary reported results. IPW weights (area under the curve [AUC], 0.658) are from logistic model of follow-up probability. Weights were derived from a logistic regression model of follow-up probability including TBI severity, Kampala Trauma Score (continuous), intensive care unit admission, and the presence of subdural hematoma, epidural hematoma, and diffuse axonal injury, each coded present or absent (AUC, 0.658). Weights were scaled ×10 before pseudo-population replication to preserve weight variation (SD, 0.10). Moderate TBI was upweighted (mean replication factor, 10.6) to represent patients with lower-severity TBI who were lost to follow-up (eTable 2 in Supplement 2).

Fewer patients with moderate TBI than severe TBI died in the hospital (22.9% [80 of 349] vs 68.9% [295 of 428]) (Table 2). Kaplan-Meier curves for inpatient cumulative mortality by TBI severity are presented in eFigure 3 in Supplement 1. Most patients died during hospitalization (48.3% [375 of 777]), with a further 16.5% (56 of 339) dying within 1 month of hospital discharge (Table 2). Cumulative mortality for moderate and severe TBI at each interval was plotted using Turnbull NPMLE curves (Figure 1). IPW-corrected and primary cumulative mortality estimates differed by 1.1 percentage points or less (eTable 2 in Supplement 1). Best-case and worst-case sensitivity bounds for cumulative mortality in moderate TBI ranged from 37.5% to 60.0% at 12 months. For patients discharged alive, 8.2% with moderate TBI (30 of 368) and 5.8% with severe TBI (26 of 451) died within 1 month. The 12-month cumulative mortality for msTBI was 75.2%: 87.8% for severe TBI and 59.4% for moderate TBI (Table 2).

**Table 2.** Patient Outcomes at the Time of Discharge and Follow-Up

Characteristic	Patients, No./total No. (%)			*P* value
	Total (N = 819)	Moderate TBI (n = 368)	Severe TBI (n = 451)	
Inpatient				
Discharge home	380/777 (48.9)	263/349 (75.4)	117/428 (27.3)	<.001
Discharged to other facility	5/777 (0.6)	0	5/428 (1.2)	
Dead	375/777 (48.3)	80/349 (22.9)	295/428 (68.9)	
Left against medical advice	17/777 (2.2)	6/349 (1.7)	11/428 (2.6)	
Unknown	42	19	23	
At 1 mo				
Alive	283/819 (34.6)	195/368 (53.0)	88/451 (19.5)	<.001
Dead	56/819 (6.8)	30/368 (8.2)	26/451 (5.8)	
Not eligible	375/819 (45.8)	80/368 (21.7)	295/451 (65.4)	
Lost to follow-up	105/819 (12.8)	63/368 (17.1)	42/451 (9.3)	
At 3 mo				
Alive	262/819 (32.0)	184/368 (50.0)	78/451 (17.3)	<.001
Dead	11/819 (1.3)	6/368 (1.6)	5/451 (1.1)	
Not eligible	431/819 (52.6)	110/368 (29.9)	321/451 (71.1)	
Lost to follow-up	115/819 (14.0)	68/368 (18.5)	47/451 (10.4)	
At 6 mo				
Alive	225/819 (27.5)	161/368 (43.8)	64/451 (14.2)	<.001
Dead	10/819 (1.2)	6/368 (1.6)	4/451 (0.9)	
Not eligible	442/819 (54.0)	116/368 (31.5)	326/451 (72.3)	
Lost to follow-up	142/819 (17.3)	85/368 (23.1)	57/451 (12.6)	
At 12 mo				
Alive	199/819 (24.3)	145/368 (39.4)	54/451 (12.0)	<.001
Dead	10/819 (1.2)	4/368 (1.1)	6/451 (1.3)	
Not eligible	452/819 (55.2)	122/368 (33.2)	330/451 (73.2)	
Lost to follow-up	158/819 (19.3)	97/368 (26.4)	61/451 (13.5)	
Follow-up mortality				
At 1 mo	56/339 (16.5)	30/225 (13.3)	26/114 (22.8)	<.001
At 3 mo	67/329 (20.4)	36/320 (16.4)	31/109 (28.4)	
At 6 mo	77/302 (25.5)	42/203 (20.7)	35/99 (35.4)	
At 12 mo	87/286 (30.4)	46/191 (24.1)	41/95 (43.2)	
Point mortality				
At 1 mo	56/339 (16.5)	30/225 (13.3)	26/114 (22.8)	<.001
At 3 mo	11/273 (4.0)	6/190 (3.2)	5/83 (6.0)	
At 6 mo	10/235 (4.3)	6/167 (3.6)	4/68 (5.9)	
At 12 mo	10/209 (4.8)	4/149 (2.7)	6/60 (10.0)	
Cumulative mortality, %^a^				
At 1 mo	58.3	36.7	75.0	
At 3 mo	64.9	46.9	79.7	
At 6 mo	67.4	48.7	82.4	
At 12 mo	75.2	59.4	87.8	

Abbreviation: TBI, traumatic brain injury.

^a^ Cumulative mortality estimates are derived from Turnbull nonparametric maximum likelihood estimation within an interval-censored framework. Estimates represent cumulative mortality from hospital admission through each time point, incorporating both in-hospital and postdischarge deaths.

Factors associated with patient mortality are presented in Table 3. Each additional year of age was independently associated with higher mortality across all 3 models (inpatient mortality: adjusted HR [AHR], 1.01 [95% CI, 1.01-1.02]; postdischarge mortality: AHR, 1.02 [95% CI, 1.01-1.03]; 12-month cumulative mortality: AHR, 1.01 [95% CI, 1.01-1.02]). In the postdischarge mortality model, severe TBI was associated with 1.62 times the hazard of mortality compared with moderate TBI (AHR, 1.62 [95% CI, 1.24-2.11]). In the 12-month cumulative mortality model, severe TBI was associated with 2.67 times the hazard of mortality compared with moderate TBI (AHR, 2.67 [95% CI, 2.24-3.18]). Patients not receiving surgical interventions had 1.64 times the hazard of inpatient mortality (AHR, 1.64 [95% CI, 1.27-2.11]) and 1.43 times the hazard of 12-month cumulative mortality (AHR, 1.43 [95% CI, 1.18-1.74]). Sensitivity analysis comparing primary and IPW-corrected AHRs is presented in eTable 3 in Supplement 1.

**Table 3.** Unadjusted and Adjusted Hazard Ratios for Inpatient, Postdischarge, and Cumulative 12-Month Mortality Among Patients With Moderate to Severe TBI

Characteristic	Hazard ratio (95% CI)^a^					
	Inpatient mortality		Postdischarge mortality		12-mo Cumulative mortality	
	Unadjusted	Adjusted	Unadjusted	Adjusted	Unadjusted	Adjusted
Age (per additional year)	1.01 (1.01-1.02)	1.01 (1.01-1.02)	1.02 (1.01-1.02)	1.02 (1.01-1.03)	1.01 (1.01-1.02)	1.01 (1.01-1.02)
Sex						
Male	1 [Reference]	1 [Reference]	1 [Reference]	1 [Reference]	1 [Reference]	1 [Reference]
Female	1.15 (0.89-1.49)	1.05 (0.80-1.37)	1.30 (0.91-1.87)	1.20 (0.85-1.69)	1.28 (1.05-1.57)	1.15 (0.93-1.42)
TBI severity						
Moderate	1 [Reference]	1 [Reference]	1 [Reference]	1 [Reference]	1 [Reference]	1 [Reference]
Severe	2.89 (2.26-3.71)	Stratified	1.54 (1.19-2.01)	1.62 (1.24-2.11)	2.56 (2.16-3.02)	2.67 (2.24-3.18)
Surgical intervention						
Yes	1 [Reference]	1 [Reference]	1 [Reference]	1 [Reference]	1 [Reference]	1 [Reference]
No	1.61 (1.26-2.07)	1.64 (1.27-2.11)	1.37 (1.01-1.87)	1.36 (0.99-1.86)	1.37 (1.13-1.67)	1.43 (1.18-1.74)
Comorbidity						
No	1 [Reference]	1 [Reference]	1 [Reference]	1 [Reference]	1 [Reference]	1 [Reference]
Yes	1.30 (1.03-1.63)	1.01 (0.77-1.32)	1.39 (1.02-1.91)	0.98 (0.67-1.43)	1.46 (1.22-1.76)	1.08 (0.86-1.37)
MSI category^b^						
Normal	1 [Reference]	1 [Reference]	1 [Reference]	1 [Reference]	1 [Reference]	1 [Reference]
Low	1.38 (1.02-1.85)	1.44 (1.06-1.94)	0.85 (0.53-1.35)	0.96 (0.60-1.52)	1.16 (0.89-1.52)	1.31 (1.01-1.71)
Borderline	0.77 (0.50-1.19)	0.86 (0.55-1.34)	0.60 (0.33-1.09)	0.73 (0.39-1.34)	0.84 (0.57-1.24)	0.94 (0.62-1.41)
High risk	0.79 (0.42-1.49)	0.81 (0.43-1.54)			0.97 (0.49-1.90)	0.95 (0.49-1.82)

Abbreviations: MSI, Modified Shock Index; TBI, traumatic brain injury.

^a^ Inpatient mortality calculated via Cox proportional hazards regression; TBI severity stratified due to proportional hazards assumption violation (Schoenfeld residuals, *P* < .001). Postdischarge and cumulative 12-month mortality calculated via interval-censored semiparametric proportional hazards regression (ic_sp, icenReg, R); standard errors obtained via 1000 bootstrap samples.

^b^ MSI categories of borderline high risk (1.31-1.69) and high risk (≥1.70) were combined to elevated risk (≥1.31) in the postdischarge mortality model only, due to sparse postdischarge events in these categories. The original 4-level MSI was retained in the inpatient mortality model and for 12-month cumulative mortality.

eTable 1 in Supplement 1 outlines the sociodemographic and clinical characteristic differences between patients who completed follow-up (n = 661) and those who did not (n = 158). Groups differed in TBI severity and KTS, with patients with lower-severity TBI disproportionately lost to follow-up.

**Figure 2.**
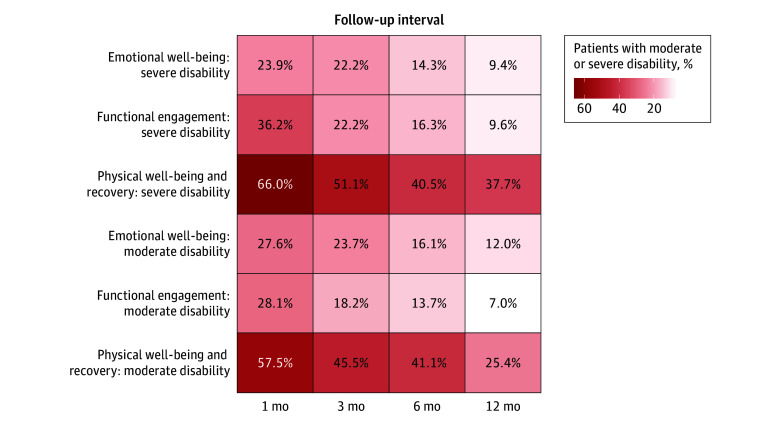
Heat Map Showing Moderate to Severe Disability in Revised Trauma-Specific Quality-of-Life Domains for Patients With Moderate to Severe Traumatic Brain Injury (TBI) at 1, 3, 6, and 12 Months Heat map showing the percentage of patients with moderate and severe disability across emotional well-being, functional engagement, and physical well-being and recovery domains at 1, 3, 6, and 12 months of follow-up. Darker shading indicates a higher proportion of disability. Results are among patients alive at 12 months.

Figure 2 represents the trajectory of moderate to severe disability in QOL domains among patients with msTBI. There was improvement in disability throughout follow-up (eTable 4 in Supplement 1). For physical well-being, a small percentage of patients with moderate TBI and patients with severe TBI initially reported no symptoms at 1 month (moderate, 12.7% [17 of 134]; severe, 10.6% [5 of 47]). However, more patients with moderate TBI than severe TBI recovered and showed no symptoms by 12 months (moderate, 21.8% [31 of 142]; severe, 15.1% [8 of 53]). Regarding functional engagement, more than 50% of the patients in both groups showed no symptoms at 1 month, with a higher rate in the moderate TBI group (moderate, 66.7% [90 of 135]; severe, 55.3% [26 of 47]). By 12 months, 88.7% of patients with moderate TBI (126 of 142) and 86.5% of patients with severe TBI (45 of 52) showed no symptoms. Regarding emotional well-being, fewer patients with moderate TBI than severe TBI had no symptoms at 1 month (38.1% [51 of 134] vs 43.5% [20 of 46]). However, symptom reduction was observed over time in both groups and peaked at 6 months for patients with severe TBI (69.1% [29 of 42]) and at 12 months for those with moderate TBI (66.2% [94 of 142]).

## Discussion

Our study analyzed 819 patients with msTBI admitted to 2 major tertiary care centers in Karachi, Pakistan. We measured short-term and long-term mortality along with QOL at each follow-up. Our study reports significantly high inpatient mortality for moderate TBI and 12-month cumulative mortality for severe TBI. Older age, having no surgical intervention, and severe injury were significantly associated with higher mortality rates both in hospital and during follow-up. Regarding QOL, the best recovery was observed in the functional engagement domain, with 88.7% of patients with moderate TBI and 86.5% of patients with severe TBI achieving complete recovery by 12 months, and the least improvement was seen in the in physical well-being and recovery domain (moderate TBI, 21.8%; severe TBI, 15.1%).

Inpatient mortality rates were 22.9% for moderate TBI and 68.9% for severe TBI. The inpatient mortality rate for moderate TBI in our study was significantly higher than inpatient mortality rates in HICs such as the US (7%)^13^ and Japan (13%),^14^ as well as other LMICs such as Afghanistan (13.9%)^15^ and India (13%).^16^ Similarly, the inpatient mortality rate for severe TBI in our study was 2 to 3 times higher than in HICs (25.1% [95% CI, 19.9%-30.2%]) and LMICs (38.3% [95% CI, 23.8%-52.7%]) reported in a recent meta-analysis, highlighting our significantly high mortality rates, even for an LMIC.^6^ These findings align with studies from Pakistan suggesting systemic constraints in TBI care.^17^

High mortality likely reflects delays in both prehospital and hospital care. Evidence from LMICs links inadequacies in emergency medical services, poor referral systems, and long travel distances to such delays.^18^ The Excellence in Prehospital Injury Care study (EPIC) reported that early management of hypoxia and hypotension and hyperventilation prevention by emergency medical services was associated with a doubling of the survival of patients with severe TBI.^19^ Evidence from Pakistan suggests that prehospital systems are often fragmented, with limited coverage, variable paramedic training, and inadequate equipment, contributing to delays in time-sensitive care.^20^ In-hospital factors, including lack of standardized trauma systems, limited critical care capacity, lack of trained emergency physicians, and delays in imaging and intervention, have also been described as factors associated with outcomes.^20^

Our study reports a 12-month cumulative mortality rate of 75.2% for msTBI, with high mortality rates among patients with severe TBI (87.8%) and those with moderate TBI (59.4%). In comparison, McCrea et al^5^ reported 12-month mortality rates of 9% for moderate TBI and 30% for severe TBI, equating to a rate more than 6 times higher in our moderate TBI cohort and 3 times higher in our severe TBI cohort. In LMIC settings, there is a dearth of literature on long-term msTBI outcomes, especially at 12 months. Some studies have recorded 6-month outcomes, but these significantly vary by setting; for example, De Silva et al^21^ reported no significant difference in 6-month moderate TBI mortality between HICs and LMICs, while Samanamalee at el^22^ reported that 42% of patients with moderate TBI were either dead or in a vegetative state, findings almost similar to those of patients with severe TBI. The CRASH (Corticosteroid Randomization After Significant Head Injury) trial data revealed that patients in LMICs with severe TBI were associated with twice the odds of dying compared with patients in HICs.^21^ Lack of proper care in the immediate postdischarge period and in rehabilitation services might be the most important factor associated with high follow-up mortality.^23,24^ This is also highlighted by the highest mortality rate at 1-month follow-up for both moderate and severe TBI. In addition, gaps in postacute and rehabilitative care have been reported in similar settings, potentially contributing to the high early postdischarge mortality observed in our cohort.^20^

Our study includes patient-reported QOL using the RT-QOL instrument, which has demonstrated reliability comparable with that of other established tools (Cronbach α ≥ 0.8). QOL across all domains was poor for patients with severe TBI compared with those with moderate TBI. The highest rate of recovery of QOL was observed in the functional engagement domain, with over 85% of surviving patients exhibiting no disabilities in activities of daily living by 12 months. This rate of functional recovery was even higher than those reported by McCrea et al,^5^ using US-based TBI data. This finding might be due to survival bias, as approximately 6 in 10 patients with moderate TBI and approximately 9 in 10 patients with severe TBI were dead by 12 months. Compared with the functional engagement domain, the physical well-being and recovery domain exhibited the least improvement, with only approximately 22% of surviving patients with moderate TBI and 15% with severe TBI achieving full recovery by 12 months. This might be associated with a lack of appropriate rehabilitation services in low-resource settings.

Sensitivity analysis showed that patients who completed follow-up were more likely than those lost to follow-up to have severe TBI. This finding is expected, as patients with severe injuries were more likely to die and not be lost to follow-up. Reasons for unsuccessful follow-up included incorrect telephone numbers, no response to calls, and a lack of telephone service or signal in the patient’s area.

Our findings have significant clinical implications. Being the first study in LMICs reporting long-term mortality among patients with msTBI, it underscores the TBI burden in both acute and chronic phases in low-resource settings, with only 1 in 10 patients with severe TBI surviving beyond 12 months. These findings show that there is a need to identify interventions to improve TBI outcomes. Our study also identified patient-specific factors (age, no surgical intervention, and severe injury) associated with poor outcomes. Early and appropriate management, along with close monitoring of these high-risk populations, could improve survival of patients with msTBI.

There is also a need to improve follow-up care, especially in the early postdischarge period, because among the total deaths in the 12-month follow-up period, 56 patients died within 1 month of hospital discharge. The system must schedule early and regular follow-ups during the immediate postdischarge period. Furthermore, strategies such as teleconsultation may be used to conduct follow-up, reducing the transportation cost burden on patients and improving follow-up compliance. Our findings also report significant residual physical disability, signifying the need for establishing rehabilitation services for patients with TBI.

### Limitations

Our study has several limitations. Both trauma hospitals are located in Karachi, so data from peripheral centers, which may have fewer resources, were not captured. Patients were recruited exclusively from neurosurgical wards, potentially introducing selection bias. The proportional hazards assumption for TBI severity in the cumulative mortality model could not be corrected within the interval-censored framework; Turnbull nonparametric curves are therefore the primary basis for severity comparisons, and the HR should be interpreted as a time-averaged approximation. Loss to follow-up was nonrandom and may have led to a modest overestimation of moderate TBI cumulative mortality despite IPW correction; best-case and worst-case sensitivity bounds ranged from 37.5% to 60.0% at 12 months. Furthermore, patient-reported QOL at each interval was calculated only for individuals who were alive at that time point. Consequently, performing complete-case analysis at each interval may have introduced additional selection bias.

## Conclusions

In this cohort study of patients with msTBI, mortality was 2 times higher than comparisons in low-resource settings and 3 times higher than comparisons in high-resource settings. Although most deaths occurred during the acute hospital stay, most postdischarge deaths occurred within the first month. Our findings emphasize the need for improved management of the acute and subacute phases, both within the hospital and during the immediate postdischarge period. An early and regular follow-up system, including potential use of telemedicine, might improve postdischarge outcomes. Further trials are required to develop evidence-based guidelines adapted to local context and assess the feasibility and effectiveness of implementing them in low-resource settings.

## Notes

Supplement 1. **eFigure 1.** Schematic Diagram of Patient Inclusion **eFigure 2.** Cumulative Mortality Sensitivity Analysis: Best-Case, Worst-Case, Primary, and IPW-Corrected Estimates by TBI Severity **eFigure 3.** Kaplan–Meier Curves for Inpatient Mortality Stratified by TBI Severity Cumulative Mortality Sensitivity Analysis: Best-Case, Worst-Case, Primary, and IPW-Corrected Estimates by TBI Severity **eTable 1.** Comparison of Baseline Epidemiological and Clinical Characteristics Between Followed-up and Lost-to-Follow-Up Patients With Moderate-Severe Traumatic Brain Injury (TBI) **eTable 2.** Cumulative Mortality (%) by TBI Severity, Follow-Up Interval, and Sensitivity Scenario **eTable 3.** Sensitivity Analysis: Primary vs Inverse Probability Weighted (IPW)-Corrected Adjusted Hazard Ratios for Postdischarge and Cumulative 12-Month Mortality **eTable 4.** Distribution of Quality-of-Life Characteristics in Trauma Patients at 1-, 3-, 6-, and 12-Month Follow-Up

Supplement 2.
Data Sharing Statement
