# Supplementary material for: Outcomes of Moderate-to-Severe Traumatic Brain Injury in a Low-Resource Setting
Source: JAMA Netw Open. 2026 Aug 11;9(8):e2628403. doi: 10.1001/jamanetworkopen.2026.28403 (PMC13463165; doi:10.1001/jamanetworkopen.2026.28403)
Supplement: Supplementary file 1 [file jamanetwopen-e2628403-s001.pdf]

Supplementary Online Content

Kumar K, Motwani S, Abdul Rahim K, et al. Outcomes of moderate-to-severe traumatic brain injury in a low-resource setting. *JAMA Netw Open.* 2026;9(8):e2628403. doi:10.1001/jamanetworkopen.2026.28403

eFigure 1. Schematic Diagram of Patient Inclusion

eFigure 2. Cumulative Mortality Sensitivity Analysis: Best-Case, Worst-Case, Primary, and IPW-Corrected Estimates by TBI Severity

eFigure 3. Kaplan–Meier Curves for Inpatient Mortality Stratified by TBI Severity Cumulative Mortality Sensitivity Analysis: Best-Case, Worst-Case, Primary, and IPW-Corrected Estimates by TBI Severity

eTable 1. Comparison of Baseline Epidemiological and Clinical Characteristics Between Followed-up and Lost-to-Follow-up Patients With Moderate-Severe Traumatic Brain Injury (TBI)

eTable 2. Cumulative Mortality (%) by TBI Severity, Follow-up Interval, and Sensitivity Scenario

eTable 3. Sensitivity Analysis: Primary vs Inverse Probability Weighted (IPW)-Corrected Adjusted Hazard Ratios for Postdischarge and Cumulative 12-Month Mortality

eTable 4. Distribution of Quality-of-Life Characteristics in Trauma Patients at 1-, 3-, 6-, and 12-Month Follow-up

This supplementary material has been provided by the authors to give readers additional information about their work.

© 2026 Kumar K et al. *JAMA Network Open.*

eFigure 1. Schematic Diagram of Patient Inclusion

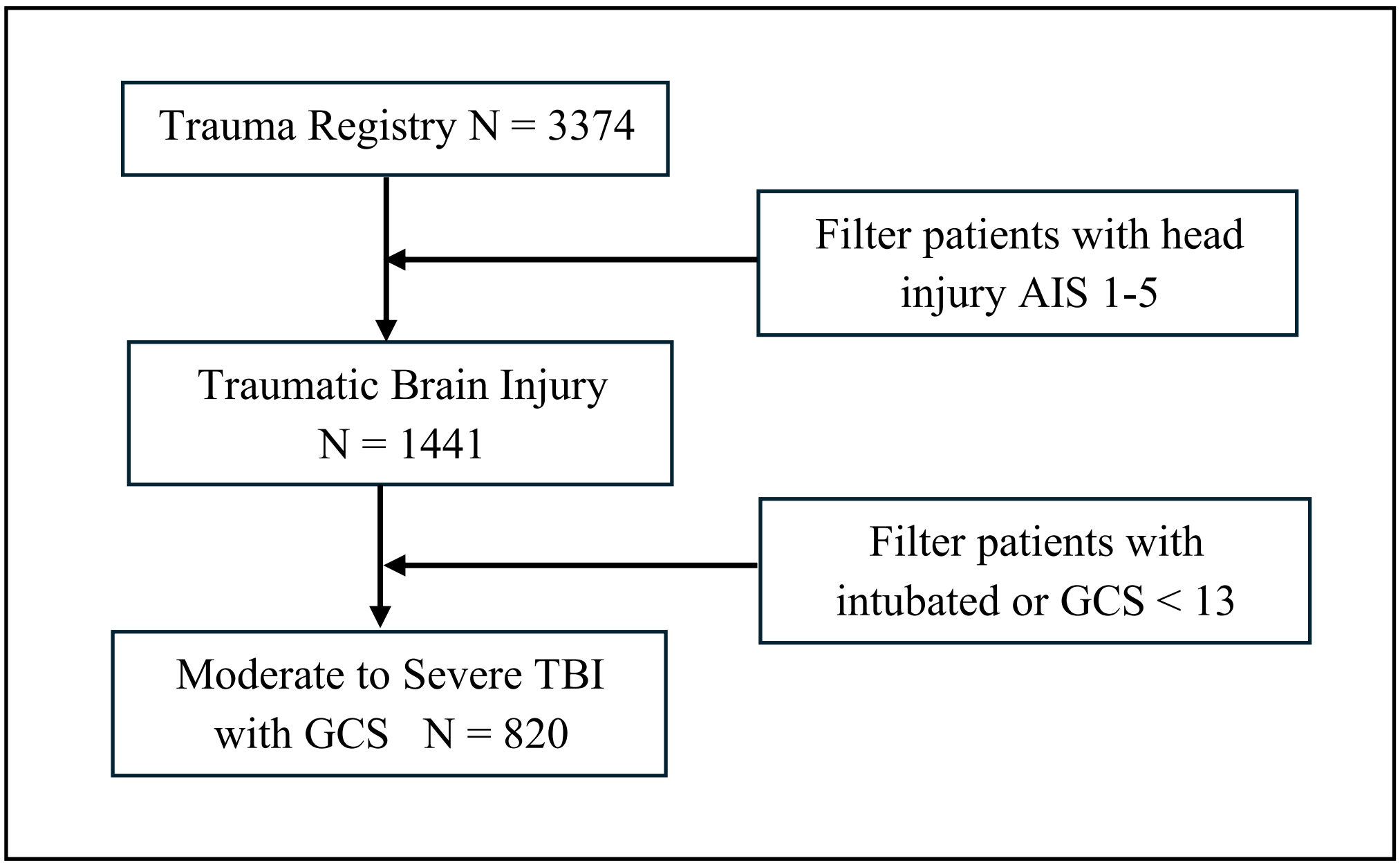

© 2026 Kumar K et al. *JAMA Network Open*

eFigure 2. Cumulative Mortality Sensitivity Analysis: Best-Case, Worst-Case, Primary, and IPW-Corrected Estimates by TBI Severity

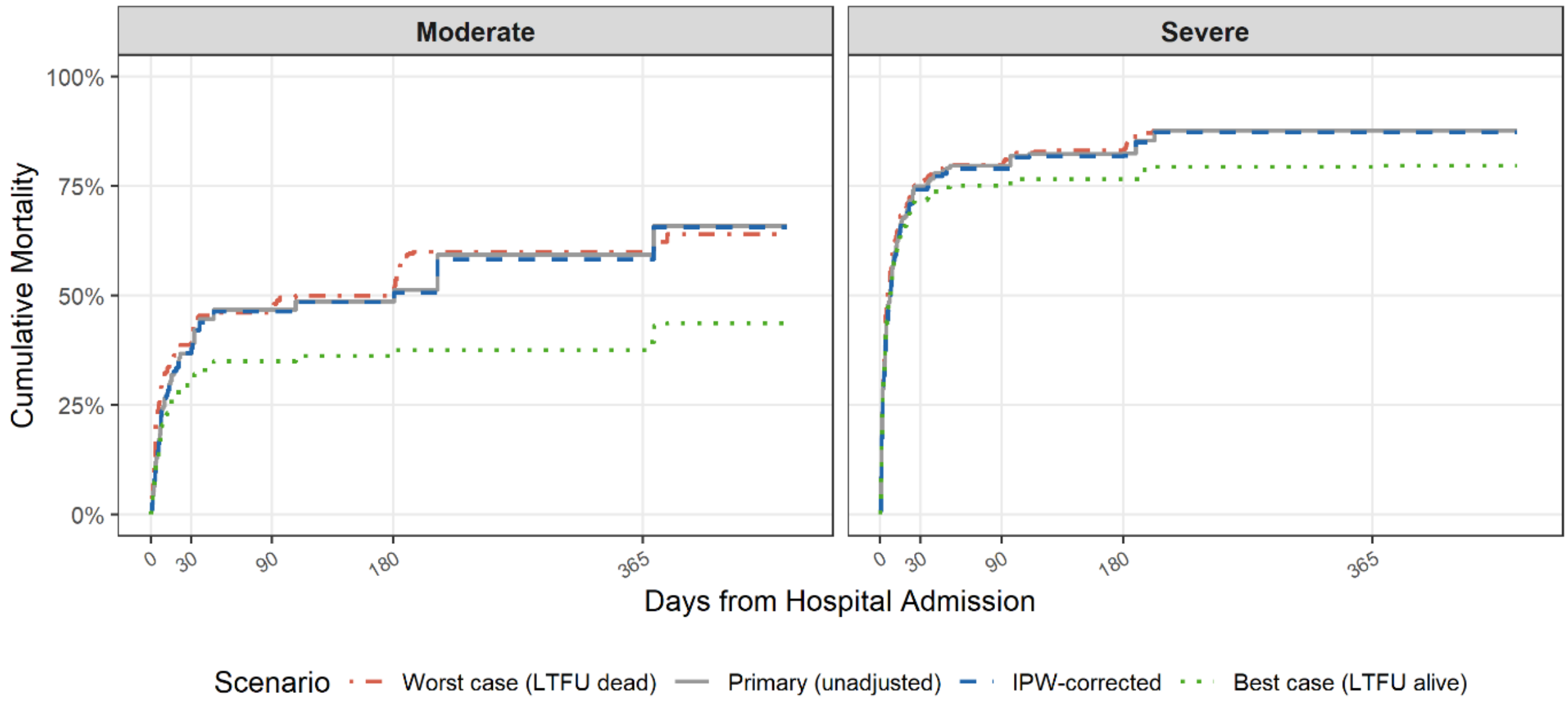

© 2026 Kumar K et al. *JAMA Network Open*

eFigure 3. Kaplan–Meier Curves for Inpatient Mortality Stratified by TBI Severity Cumulative Mortality Sensitivity Analysis: Best-Case, Worst-Case, Primary, and IPW-Corrected Estimates by TBI Severity

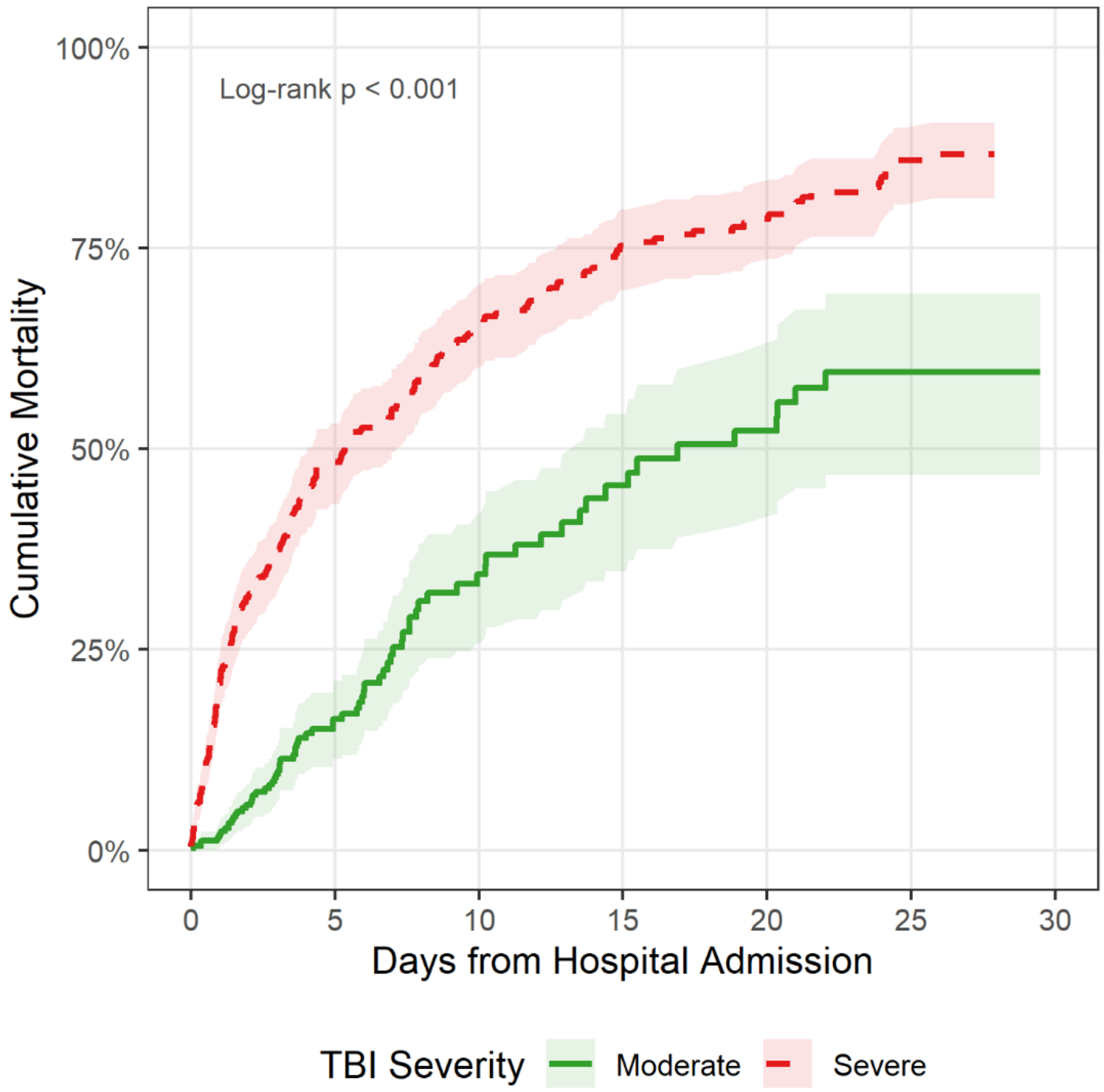

© 2026 Kumar K et al. *JAMA Network Open*

eTable 1. Comparison of Baseline Epidemiological and Clinical Characteristics Between Followed-up and Lost-to-Follow-up Patients With Moderate-Severe Traumatic Brain Injury (TBI)

**Characteristics**	**Followed Up**<br>**N = 661**	**Loss to Follow-up**<br>**N = 158**	**P value**
**Age group**			0.06
18-39	326/661 (49.32)	94/158 (59.49)	
40-64	252/661 (38.12)	51/158 (32.28)	
≥ 65	83/661 (12.56)	13/158 (8.23)	
**Gender**			0.20
Male	561/661 (84.87)	128/158 (81.01)	
Female	100/661 (15.13)	30/158 (18.99)	
**Educational Status**			>.99
No Formal Education	237/656 (36.13)	58/157 (36.94)	
Primary Education	132/656 (20.12)	30/157 (19.11)	
Secondary Education	159/656 (24.24)	38/157 (24.20)	
Intermediate	69/656 (10.52)	18/157 (11.46)	
Graduation and above	59/656 (8.99)	13/157 (8.28)	
Unknown	5	1	
**Cause of Injury**			0.60
RTC	485/661 (73.37)	122/158 (77.22)	
Fall	138/661 (20.88)	26/158 (16.46)	
Assault	22/661 (3.33)	5/158 (3.16)	
Other	16/661 (2.42)	5/158 (3.16)	
**RTC victim type**			>0.99
Passenger	88/475 (18.53)	23/118 (19.49)	
Rider (Motorcycle)	298/475 (62.74)	72/118 (61.02)	
Driver (Car)	9/475 (1.89)	2/118 (1.69)	
Pedestrian	80/475 (16.84)	21/118 (17.80)	
Unknown	186	40	
**KTS**			<0.001
Mild	155/654 (23.70)	64/157 (40.76)	
Moderate	389/654 (59.48)	82/157 (52.23)	
Severe	110/654 (16.82)	11/157 (7.01)	
Unknown	7	1	
**Hospital Ward**			0.008
HDU	509/638 (79.78)	134/148 (90.54)	
ICU	111/638 (17.40)	11/148 (7.43)	
Ward	18/638 (2.82)	3/148 (2.03)	
Unknown	23	10	
**Surgical Intervention**			0.09
No	488/661 (73.83)	127/158 (80.38)	
Yes	173/661 (26.17)	31/158 (19.62)	
**TBI severity**			< 0.001
Moderate	271/661 (41.00)	97/158 (61.39)	
Severe	390/661 (59.00)	61/158 (38.6)	
**Procedure**			0.61
Craniotomy	68/173 (39.31)	15/31 (48.39)	
Craniectomy	48/173 (27.75)	7/31 (22.58)	
Burr hole	22/173 (12.72)	3/31 (9.68)	
**TBI Diagnosis**			
Contusions	269/661 (40.69)	67/661 (42.40)	0.60
Subdural Hematoma	258/661 (39.03)	39/661 (24.68)	0.002
Epidural Hematoma	122/661 (18.45)	43/661 (27.21)	0.009
Skull Fracture	78/661 (11.80)	16/661 (10.12)	0.76

© 2026 Kumar K et al. *JAMA Network Open*

Intracerebral haemorrhage	78/661 (11.80)	11/661 (6.96)	0.08
Diffuse Axonal Injury	30/661 (4.53)	13/661 (8.22)	0.03
Pneumocranium	8/661 (1.21)	5/661 (3.16)	0.27

eTable 2. Cumulative Mortality (%) by TBI Severity, Follow-up Interval, and Sensitivity Scenario

**TBI Severity**	**Scenario**	**1 month**	**3 months**	**6 months**	**12 months**
Moderate	Worst case (LTFU assumed dead)	39.20%	46.10%	50.40%	60.00%
Moderate	Primary (unadjusted)	36.70%	46.90%	48.70%	59.40%
Moderate	IPW-corrected	36.90%	46.50%	48.60%	58.30%
Moderate	Best case (LTFU assumed alive)	29.60%	35.00%	36.20%	37.50%
Severe	Worst case (LTFU assumed dead)	75.6%	79.8%	83.5%	87.8%
Severe	Primary (unadjusted)	75.0%	79.7%	82.4%	87.8%
Severe	IPW-corrected	74.2%	79.0%	81.9%	87.4%
Severe	Best case (LTFU assumed alive)	71.8%	75.2%	76.6%	79.4%

© 2026 Kumar K et al. *JAMA Network Open*

**eTable 3.** Sensitivity Analysis: Primary vs Inverse Probability Weighted (IPW)-Corrected Adjusted Hazard Ratios for Postdischarge and Cumulative 12-Month Mortality						
**Characteristic**						
	**Primary aHR (95% CI)**	**IPW aHR (95% CI)**	**Δ(pp)[a]**	**Primary aHR (95% CI)**	**IPW aHR (95% CI)**	**Δ (pp)[a]**
**Age** (per year, continuous)	1.02 (1.01–1.03)	1.02 (1.01–1.03)	+0.00	1.01 (1.01–1.02)	1.01 (1.01–1.02)	+0.00
**Gender**						
Male						
Female	1.20 (0.85–1.69)	1.18 (0.84–1.67)	-0.02	1.15 (0.93–1.42)	1.15 (0.92–1.44)	+0.00
**TBI severity**						
Moderate						
Severe	1.62 (1.24–2.11)	1.70 (1.30–2.23)	+0.08	2.67 (2.24–3.18)	2.66 (2.23–3.18)	-0.01
**Surgical Intervention**						
Yes						
No	1.36 (0.99–1.86)	1.35 (0.98–1.84)	-0.01	1.43 (1.18–1.74)	1.42 (1.17–1.74)	-0.01
**Comorbidity**						
No						
Yes	0.98 (0.67–1.43)	1.00 (0.68–1.46)	+0.02	1.08 (0.86–1.37)	1.10 (0.87–1.40)	+0.02
**Modified Shock Index**						
Normal						
Low	0.96 (0.60–1.52)	0.94 (0.59–1.50)	-0.02	1.31 (1.01–1.71)	1.29 (0.99–1.70)	-0.02
Elevated	0.73 (0.39–1.34)	0.73 (0.39–1.35)	+0.00	-	-	-
Borderline	-	-	-	0.94 (0.62–1.41)	0.93 (0.62–1.40)	-0.01
High risk	-	-	-	0.95 (0.49–1.82)	0.99 (0.50–1.99)	+0.04

[a]Δ (pp): difference in hazard ratio between IPW-corrected and primary estimate (IPW minus primary). Cells highlighted in pale yellow indicate a difference ≥0.05 HR units. Positive values indicate the IPW estimate is higher than the primary; negative values indicate lower.

[b]MSI Borderline (1.31–1.69) and High Risk (≥1.70) collapsed to Elevated (≥1.31) in the Follow-up mortality model due to sparse post-discharge events. Original four-level MSI retained in the Cumulative Mortality model

© 2026 Kumar K et al. *JAMA Network Open*

eTable 4. Distribution of Quality-of-Life Characteristics in Trauma Patients at 1-, 3-, 6-, and 12-Month Follow-up

Characteristics	1 Month		3 Months		6 Months		12 Months	
	Moderate	Severe	Moderate	Severe	Moderate	Severe	Moderate	Severe
**Physical Well-being and Recovery**								
**No symptom**	17 (12.69)	5 (10.64)	26 (19.70)	8 (17.78)	18 (14.52)	6 (14.29)	31 (21.83)	8 (15.09)
**Mild**	40 (29.85)	11 (23.40)	46 (34.85)	14 (31.11)	55 (44.35)	19 (45.24)	75 (52.82)	25 (47.17)
**Moderate**	38 (28.36)	16 (34.04)	37 (28.03)	14 (31.11)	34 (27.42)	12 (28.57)	22 (15.49)	16 (30.19)
**Severe**	39 (29.10)	15 (31.91)	23 (17.42)	9 (20.00)	17 (13.71)	5 (11.90)	14 (9.86)	4 (7.55)
**No opinion**	11	7	13	9	21	12	3	1
**Functional Engagement**								
**No symptom**	90 (66.67)	26 (55.32)	103 (78.03)	33 (73.33)	104 (83.87)	33 (76.74)	126 (88.73)	45 (86.54)
**Mild**	7 (5.19)	4 (8.51)	5 (3.79)	2 (4.44)	3 (2.42)	3 (6.98)	6 (4.23)	2 (3.85)
**Moderate**	6 (4.44)	3 (6.38)	4 (3.03)	3 (6.67)	3 (2.42)	1 (2.33)	3 (2.11)	1 (1.92)
**Severe**	32 (23.70)	14 (29.79)	20 (15.15)	7 (15.56)	14 (11.29)	6 (13.95)	7 (4.93)	4 (7.69)
**No opinion**	10	7	13	9	21	11	3	2
**Emotional Well-being**								
**No symptom**	51 (38.06)	20 (43.48)	65 (49.62)	21 (46.67)	79 (63.71)	29 (69.05)	94 (66.20)	32 (60.38)
**Mild**	46 (34.33)	15 (32.61)	35 (26.72)	14 (31.11)	25 (20.16)	7 (16.67)	31 (21.83)	16 (30.19)
**Moderate**	26 (19.40)	8 (17.39)	23 (17.56)	9 (20.00)	13 (10.48)	6 (14.29)	13 (9.15)	4 (7.55)
**Severe**	11 (8.21)	3 (6.52)	8 (6.11)	1 (2.22)	7 (5.65)	0 (0.00)	4 (2.82)	1 (1.89)
**No opinion**	11	8	14	9	21	12	3	1

© 2026 Kumar K et al. *JAMA Network Open*
