# Supplementary material for: Outcomes of Moderate-to-Severe Traumatic Brain Injury in a Low-Resource Setting
Source: JAMA Netw Open. 2026 Aug 11;9(8):e2628403. doi: 10.1001/jamanetworkopen.2026.28403 (PMC13463165; doi:10.1001/jamanetworkopen.2026.28403)
Supplement: Supplementary file 2 [file jamanetwopen-e2628403-s002.pdf]

Data Sharing Statement

Kumar. Outcomes of Moderate-to-Severe Traumatic Brain Injury in a Low-Resource Setting. *JAMA Netw Open*. Published August 11, 2026. doi:10.1001/jamanetworkopen.2026.28403

Data
Data available: No

Additional Information
Explanation for why data not available: This is an institutional data which would be available from corresponding author at a reasonable request.
